# Making Sense of Infant Familiarity and Novelty Responses to Words at Lexical Onset

**DOI:** 10.3389/fpsyg.2016.00715

**Published:** 2016-05-18

**Authors:** Rory A. DePaolis, Tamar Keren-Portnoy, Marilyn Vihman

**Affiliations:** ^1^Communication Sciences and Disorders, James Madison University, HarrisonburgVA, USA; ^2^Language and Linguistic Science, University of YorkYork, UK

**Keywords:** novelty, familiarity, early word learning, headturn preference procedure, infant speech perception

## Abstract

This study suggests that familiarity and novelty preferences in infant experimental tasks can in some instances be interpreted together as a single indicator of language advance. We provide evidence to support this idea based on our use of the auditory headturn preference paradigm to record responses to words likely to be either familiar or unfamiliar to infants. Fifty-nine 10-month-old infants were tested. The task elicited mixed preferences: familiarity (longer average looks to the words likely to be familiar to the infants), novelty (longer average looks to the words likely to be unfamiliar) and no-preference (similar-length of looks to both type of words). The infants who exhibited either a familiarity or a novelty response were more advanced on independent indices of phonetic advance than the infants who showed no preference. In addition, infants exhibiting novelty responses were more lexically advanced than either the infants who exhibited familiarity or those who showed no-preference. The results provide partial support for Hunter and Ames’ (1988) developmental model of attention in infancy and suggest caution when interpreting studies indexed to chronological age.

## Introduction

The auditory headturn preference paradigm (AHPP), which has been used since the 1980s (Fernald, 1985; Kemler Nelson et al., 1995), has been instrumental in understanding infants’ ability to process speech (for a partial review see Gerken and Aslin, 2005). The success of the paradigm is based on the exploitation of two well-established types of infant responses to stimuli, familiarity and novelty responses, expressed as enhanced attention to either familiar or novel stimuli, respectively. A familiarity response can be thought of as involving matching of stimuli to an existing partially formed memory trace, while a novelty response would occur at a more advanced stage, after the familiar stimuli have been more completely processed and an infant’s attention is free to turn to less well-represented stimuli (Rose et al., 1982; Roder et al., 2000). Typically, in a single study either a familiarity or a novelty response at the group level is taken to suggest that the infants have noticed some aspect of the stimuli. But what does it mean when both novelty and familiarity are observed in a single experiment? In this paper we argue that a mixture of familiarity and novelty responses can, under some conditions, be interpreted together as indicating developmental advance.^1^ In the process we provide an empirical test of the Hunter and Ames (1988) model of the underlying mechanisms that elicit familiarity and novelty responses, as it applies to words at the onset of a developing lexicon (see below for a detailed description of the Hunter and Ames model).

Factors that affect novelty and familiarity responses in infants have been extensively studied since the methodology was first developed (Dember and Earl, 1957; Fantz, 1958, 1964) and two factors have emerged as the primary determinants of the type of response elicited from infants. The first is the role of stimulus complexity in the progression of responses from familiarity to novelty (Cornell, 1975; Martin, 1975; Kinney and Kagan, 1976; Kaplan and Werner, 1986; Hunter and Ames, 1988; Burnham and Dodd, 1998; Roder et al., 2000). Very simple stimuli may lead to a novelty preference, whereas more complex stimuli, which necessitate more elaborate processing, may lead to a familiarity preference. However, the relationship between preference and complexity is, in a word, complex. For example, Kidd et al. (2012) familiarized infants to visual stimuli that varied from high- to low-predictability events. These investigators found that the infants looked away both from stimuli that were overly complex (low predictability) *and* from stimuli that were overly simple (high predictability). At the same time, there is also an interaction between complexity and development, since the same stimuli can elicit both a familiarity and a novelty pattern in infants of different ages (Colombo and Bundy, 1983).

The second major factor that affects infant responses to familiar or novel stimuli is familiarization time. Fantz (1964) found that infants fixated progressively less to familiar relative to novel stimuli. Since that classic finding, familiarization has been studied in detail both across and within ages (e.g., Martin, 1975; Colombo and Bundy, 1981; Rose et al., 1982; Bahrick and Pickens, 1995; Burnham and Dodd, 1998; Roder et al., 2000). In a typical experiment an infant is familiarized to a stimulus and the infant’s attention is measured relative to a similar but novel stimulus (see Rose et al., 1982, for an example). With brief familiarization time infants show a preference for the familiarized pattern, but as exposure time in the habituation phase increases, the preference shifts to the novel stimulus. What has emerged from many of these studies is a progression from familiarity to novelty that, like the notion of complexity itself, is further complicated by development. For example, Bahrick and Pickens (1995) found that, after a familiarization phase, infants showed a novelty effect after delays of only 1 min between familiarization and testing but a familiarity response after a delay of 1 month. Intermediate-length delays – between 1 min and 1 month – elicited no preference from the infants. In addition, Colombo and Bundy (1983) found that the same stimuli that elicited a familiarity response at 2 months of age elicited a novelty response at 4 months of age. Thus, complexity and familiarization time interact with development in a complex manner.

Interestingly, novelty and familiarity are often used in the same experiment to index the same behavior. For example, McMurray and Aslin (2005) exposed infants to one of two endpoints on the voice onset time (VOT) continuum between a /b/ and a /p/. Upon analyzing the results, they sub-categorized the infants into those who responded with longer looking time (LT) to the familiarized stimuli versus those who responded to the novel stimuli. They then used *both* the novelty and familiarity infants to argue for infant sensitivity to within-phonetic-category differences. McMurray and Aslin (2005, p. B20) explicitly left open the question of why infants might respond differently in this experiment, suggesting that “no consensus has emerged, and few studies make *a priori* predictions” as to why this is the case.

Thiessen et al. (2005) suggest something similar. They familiarized infants with 12 artificial-language sentences recorded with prosody that is consistent with either infant-directed or adult-directed speech (IDS or ADS). Then all of the infants heard whole- and part-word lists (part-words being syllable sequences from within the sentences that crossed word boundaries) in ADS. Only the infants who had been familiarized with the sentences in IDS exhibited a preference for the whole words (familiarity), suggesting that IDS facilitated word segmentation. Based on models predicting infant novelty versus familiarity responses (Wagner and Sakovits, 1986; Hunter and Ames, 1988) the authors reasoned that an alternative explanation for their results could be that the infants had segmented words under both IDS and ADS, but the infants exposed to ADS were faced with an easier task, as they were matching like to like (ADS at familiarization to ADS at test). This, the authors thought, may have led them to exhibit a mix of familiarity and novelty responses, resulting in a group response of no preference. The infants exposed to IDS, however, showed a familiarity preference for whole words due to their task being more difficult, involving a mismatch in speech style between the familiarization and the test stimuli. Essentially, the authors argued that stimulus complexity might be driving the results. To explore this further they made the task easier by doubling the length of the familiarization and testing slightly older infants. In the second experiment the infants in the ADS condition again showed no preference, whereas those in the IDS condition showed a novelty preference for part-words over whole words. The authors saw this as evidence that the infants in the ADS condition did not succeed in segmenting the words in either experiment. The switch in the IDS group to novelty when the task become ‘less complex,’ partly due to an increase in processing time prior to testing and partly due to the increased age of the infants, suggests that novelty and familiarity responses could be used to investigate developmental differences in infants at a single age.

Others have found both novelty and familiarity in a single study (e.g., Gerken et al., 2015), but the Thiessen et al. (2005) study highlights the importance of tracking the characteristics of the stimuli (auditory, visual, complex, bright, colorful, soft, loud, simple, ecologically relevant, etc.). It could be argued that each new type of stimulus requires a methodological rethink as to how the parameters will affect infant responses. For example, although words are often explored in novelty/familiarity paradigms either in isolation (e.g., Hallé and de Boysson-Bardies, 1994, 1996; Vihman et al., 2004; Swingley, 2005) or in passages of sentences containing target words (e.g., Jusczyk and Aslin, 1995; Jusczyk et al., 1999; Bortfeld et al., 2005; Singh, 2008; Singh et al., 2012; DePaolis et al., 2014), there are few methodological examinations of familiarity and novelty as they apply specifically to the developing lexicon. One exception is a computational model of factors affecting word segmentation in AHPP experiments (Bergmann et al., 2013). Another is a study (DePaolis et al., 2013) that found that 12-month-old infants’ preference for non-words was linearly related to the number of consonants each infant produced that were featured in the test stimuli; effectively, the infants showed either familiarity or novelty, based upon the extent of their previous practice with the test stimuli (see DePaolis et al., 2011 and Majorano et al., 2014 for similar differences in infant preference based upon their babbling patterns). The dearth of studies of novelty and familiarity as they relate to word learning is surprising, however, since using words as stimuli can introduce complex elements of associative memory that are typically not present with other stimuli, such as consonants.

Untrained words may be expected to elicit novelty, familiarity, and no preference responses from different infants of the same age but at different stages of lexical advance. Thus words should be the ideal stimulus to investigate the phenomenon of developmental stage, rather than its proxy – chronological age – since attentional responses to words will depend more upon lexical experience than age. A series of studies in Dutch, English, and French contrasting familiar versus unfamiliar word lists (Hallé and de Boysson-Bardies, 1994, 1996; Vihman et al., 2004; Swingley, 2005) suggests a paradigm well suited for this line of inquiry. Using the AHPP the experimenters determined that infants have stronger representations of word *forms* (independent of contextual cues for meaning, such as seeing or playing with a ball while hearing the word *ball*) by 11 months than at 9 months of age. The term ‘word form’ is used to indicate that this recognition need not imply understanding of the word’s meaning or reference.

This task is very different from that of identifying (or segmenting) experimentally familiarized words within running speech, a skill that may emerge as early as 7.5 months of age (Jusczyk and Aslin, 1995; Jusczyk et al., 1999, but see Floccia et al., 2016). Segmentation immediately after familiarization with a pair of words can be thought of as drawing upon short-term memory; word form recognition or segmentation without experimental familiarization, as in the word-form recognition task, must be based upon representation in long-term memory – essentially drawing upon the lexicon that is just beginning to emerge. The emphasis in the word-form recognition task on a newly formed but unstable representation of often-heard words makes it likely that, at the onset of lexical development, the task will elicit both familiarity and novelty responses, since the level of lexical advance of individual infants can be expected to vary considerably.

This variability is just what was found when the word-form recognition experiments were replicated in a cross-sectional design, at 9, 10, 11, and 12 months (Vihman et al., 2007). Of particular interest to the current study is that at 10 months of age roughly half of the infants tested exhibited a familiarity response while the other half exhibited a novelty response, with a gradient from weak to strong preferences in both directions. These results show that testing infants at 10 months of age on isolated word-form recognition yields a high degree of variability in response to the familiar words, as the test elicits both familiarity and novelty responses; thus the word-form recognition paradigm is an ideal vehicle for exploring the nature of novelty and familiarity responses in a single experiment. If the variability in responses is due to the variability in the development of a lexicon, separate measures of lexical and/or phonetic advance might be able to explain or predict the responses on the AHPP.

In this study we have adopted the AHPP paradigm and the Hunter and Ames (1988) model of infant response to stimuli in formulating our initial hypothesis that no-preference, familiarity and novelty responses can each reflect a different level of lexical advance at a single age. This type of model suggests that ‘preferences for novelty and familiarity are not tied to particular ages but instead can be found at any age, depending on the duration of previous familiarization and on task difficulty relative to the age and experience of the infant.’ (Hunter and Ames, 1988, p. 70; for similar models see Rose et al., 1982; Kaplan and Werner, 1986; Wagner and Sakovits, 1986; Bahrick and Pickens, 1995). The core idea is that an infant’s attention to a stimulus is dictated by the stability of the representation of the stimulus in memory. A hypothetical experiment supporting this type of model would familiarize an infant to a set of previously unknown words and then contrast these words with other unknown and unfamiliarised words. If the familiarization phase is too short for the infant to begin to form a tentative memory, infants will show no preference for either word list. Subsequent presentations of these words would elicit longer looks due to the newly formed memory, and continued presentation of these words would elicit a decrease in interest in the familiar words due to the increasing stability of the representation of the word in memory, thus yielding a novelty response.

**FIGURE 1:**
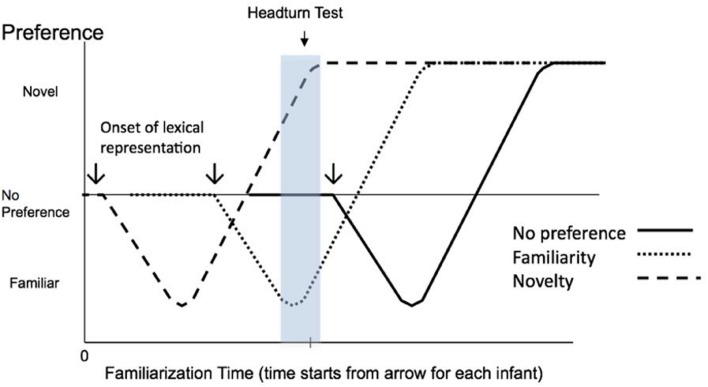
**Schematic predicted responses of three infants at different levels of lexical advance (all three figures are redrawn from Hunter and Ames, 1988).** The downward arrows indicate the onset of lexical representation for each infant. The hypothesized curve for each infant represents his or her response to lists of (untrained) familiar words contrasted with unfamiliar words. The shaded area demonstrates that at specific testing points novelty and familiarity would both indicate the recognition of word forms (dashed lines), while no preference would indicate a lack of sensitivity to word forms (solid line). The vertical axis is the strength of the familiarity or novelty effect. The horizontal axis represents the time for a novelty effect to shift to a familiarity effect. Each infant would have their own individual time scale for beginning to recognize words in everyday life, although for clarity the three infants depicted in this figure are treated as having identical time scales for the shift in word-form recognition.

In the word-form recognition experiments described above (Hallé and de Boysson-Bardies, 1994, 1996; Vihman et al., 2004, 2007; Swingley, 2005) the familiarized stimulus is the child’s own lexicon, since familiarization, if it can be called that, occurs very gradually over the course of everyday exposure to words in the period before the infant is brought to the lab. **Figure 1** displays the hypothesized development of three infants’ familiarity and novelty responses to the stimuli in a word-form recognition experiment based on the Hunter and Ames model. The figure has been redrawn from Hunter and Ames (1988) to show the progression from familiarity through to novelty as a function of length of exposure to familiar words. It tracks three hypothetical infants who begin at different ages to represent word forms independent of context, an achievement which can signal the onset of lexical representation (Swingley, 2009).

The infant characterized by the solid line has not started forming lexical representations by the time of the experiment, so that no memory trace has been formed that is strong enough to elicit either a novelty or a familiarity response to the words heard during the test that were expected to be familiar (i.e., words chosen from pooled parental reports for early vocabulary). This infant will thus most likely exhibit no preference for either the familiar or unfamiliar words. The infants characterized by the dashed lines are more lexically advanced; they have begun constructing the knowledge and skills necessary for lexical representation at the time of the test. Depending upon the speed with which each infant processes the newly learned words, the comparison of the words heard in the test to partially or well-formed memory traces of familiar words will lead to either familiarity or novelty responses, respectively.

It is also possible that the more advanced infants will fail to exhibit a preference (note the transition through *No preference* for each infant in **Figure 1**), although Roder et al. (2000) found no evidence that the transition from familiarity to novelty was mediated by a period of no preference. It is likely that if, between the time in which a familiarity and a novelty response are elicited, there is a period in which the infant displays equal interest in the stimuli, it is a very brief one, as can be seen by the steep hypothesized slope of the line in the transition between these two responses in **Figure 1**. It should also be noted, however, that there is some disagreement as to whether the Hunter and Ames model holds in every case, especially insofar as it relates to a period of no preference during the shift from familiarity to novelty (see Slater, 2004, for discussion). However, recall that the Hunter and Ames model does hypothesize a longer, more stable no-preference period prior to the onset of any learning.

Further complicating this picture is the potentially variable speed of progression from familiarity to novelty, which again depends on the speed of processing for familiar words. In **Figure 1** the three infants are plotted as having identical processing speeds, while the Roder et al. (2000) experiments suggest that the pace would likely vary by infant. Crucially, from the perspective of this study, the putative contraction or expansion of the progressions for different infants in any AHPP experiment should make it even more likely that both familiarity and novelty would be elicited by the same stimuli at the same age, since both reflect recognition of the familiar words, although probably to differing degrees.

Regardless of the processing timescale and the slope of the change in preference for familiar words, if the model depicted in **Figure 1** is sound, then some experiments should yield both a familiarity and a novelty effect and these two effects should both signify recognition of familiar words and signal that the onset of lexical representation has begun. The important point to note is that in such cases it is expected that the most advanced infants will exhibit a novelty effect while the infants who are the least advanced in terms of lexical representation will tend not to show a preference [corresponding to the early No-preference period in the Hunter and Ames (1988) model]. The infants who exhibit a familiarity effect would be predicted to fall between these two groups in terms of lexical representation. Recall that the experiments by McMurray and Aslin (2005) and Thiessen et al. (2005) support this possibility.

To validate this interpretation of the mix of preference behaviors in the AHPP separate measures of language advance are needed to justify an independent grouping of infants into more versus less advanced. This is the rationale behind the current study, which used additional measures of phonetic and lexical advance to corroborate the division of the infants, based on their AHPP responses, into three groups: two advanced groups showing a relatively strong familiarity or novelty response and a less advanced group, showing no clear preference.

The variable chosen to estimate phonetic advance was the age of attainment of two vocal motor schemes or VMS (measured by the repeated production of a specific consonant), which indicates the degree to which an infant is using a consonant consistently and repeatedly in babble (McCune and Vihman, 2001). Previous research has shown that attainment of one or more VMS, beyond indexing phonetic advance, affects the infant’s response to similar consonants in running speech (DePaolis et al., 2011). Additional work has shown that acquisition of the second VMS is a necessary step for referential word use (McCune and Vihman, 2001) or for word use in general (Keren-Portnoy et al., 2005; McGillion et al., 2016). Each of these studies points to the importance of VMS for forming stable lexical representations.

Our measure of lexical advance was the number of words that the infant is able to comprehend according to parental report, using the Oxford adaptation of the MacArthur Communicative Developmental Inventory (CDI: Hamilton et al., 2001), a widely used measure of vocabulary development. Receptive vocabulary size, as reported by parents, is also an estimate of the infant’s level of lexical representation, although it presumably includes meaning in addition to word form recognition. Interestingly, CDI estimates of lexicon size between 12 and 24 months have been shown to be correlated with performance on the AHPP (word segmentation) at 7–8 months of age (Singh et al., 2012), supporting the use of this measure to identify infants who are likely to recognize word forms in this study.

In summary, the purpose of this study was to investigate the possibility that familiarity and novelty responses both reflect the onset of lexical representation in 10-month-old infants. In order to do this we used the AHPP to run word-form recognition experiments on 59 infants at 10 months of age, a point at which we expected roughly half of the infants to recognize familiar words. At the same time, we collected independent measures of phonetic and lexical advance for those same infants. We hypothesized that these additional measures would agree with the categorisation of the infants suggested by the AHPP response. Specifically, if **Figure 1** is valid in its conceptualisation of the interplay between familiarity and novelty, the infants who exhibit either a familiarity or a novelty response should be more advanced in both phonetic and lexical ability than their peers who show no preference for either type of stimulus. In addition, we hypothesize that there will be a progression of lexical and phonetic advance, with the infants showing no preference being the least advanced, followed by those showing familiarity, and finally by those showing novelty responses, who would be the most advanced.

## Materials and Methods

### Participants

A total of 59 infants participated in a 9-month longitudinal study from which this data were taken. Infants were recruited through ads in newspapers and in local shops and playgroups. None of the participants had any known developmental or hearing problems (all infants had been screened by the National Health Service). This study was approved by the Ethics Committee of the University of York. Written informed consent was obtained from all participating families.

### Variables

#### Phonetic Advance

Age at two VMS was assessed using half-hour weekly audio and video recordings made in the home, starting at 9 months of age and continuing until the infant attained two high-use VMS (defined as supraglottal consonants only, typically a labial, alveolar or velar stop or a labial or alveolar nasal). The recordings were transcribed phonetically, and consonants used in vocalizations (mostly babble, but in some cases words as well) were tallied. A consonant was considered to have reached VMS status if it fulfilled one of two criteria: (1) A minimum of ten tokens of the given consonant were produced in each of at least three out of four consecutive half-hour sessions (McCune and Vihman, 2001) or (2) a total of 50 or more tokens of the given consonant were produced in one to three successive recording sessions (DePaolis et al., 2011). We dated the emergence of a VMS to the first of these criterial sessions. Age of attainment of the second VMS was dated to the first criterial session for the child’s second VMS.

#### Lexical Advance

Receptive lexicon size estimates were based upon the Oxford CDI (Hamilton et al., 2001), completed by parents when the infants were 9 months old and then monthly thereafter, although not every parent completed the questionnaire every month. An average of 2.5 (*SD* = 2.11) parental reports were missing per child, out of the expected 10 monthly CDIs.

#### Auditory Headturn Preference Procedure (AHPP)

The word-form recognition test was administered at 10 months. The stimuli were lists of words produced in isolation. Half of the lists consisted of 12 words likely to be familiar to the infants (Familiar words, based on CDI data from a previous study of 99 infants being raised in English in North Wales, aged 9–11 months.) The other half consisted of 12 words unlikely to be familiar to infants (Rare words, based on frequency counts of no more than 6 in 1,014,232 in Francis and Kučera, 1982). The Rare words were comparable to the Familiar words in terms of their segments (consonant and vowels) and phonotactics. (See **Table 1** for stimuli. We constructed two lists for each type of stimulus, with half the infants being presented with list A and half with list B, for both Familiar and Rare words.)

**Table 1:** Word stimuli for the AHPP experiment.

List A		List B	
	
Familiar	Rare	Familiar	Rare
Baby	Pauper	Birdie	Beadle
Biscuit	Tendon	Bottle	Blotter
Breakfast	Brindle	Clever	Dapper
Careful	Geezer	Dolly	Gully
Cuddle	Dabble	Gentle	Tendril
Mummy	Deacon	Grandad	Plunder
Dinner	Berber	Daddy	Gecko
Dirty	Turbo	Nappy	Netter
Dummy	Tinny	Naughty	Doughty
Granma	Crofter	Teddy	Tatty
Telly	Demi	Tickle	Kindle
Tired	Mired	Toothbrush	Tangram


### Procedure

#### Naturalistic Recordings

Infants were recorded at home in naturalistic play interactions with a caregiver, once a week. The recordings were made using a Sony digital video camera recorder, either HDV 1080i HVR-A1E or DSR-PDX10P. The recordings were then transferred digitally to a computer and transcribed phonetically by one of three experienced transcribers, using ELAN Linguistic Annotator. Reliability among transcribers was calculated based on four 3-min sections randomly sampled from the 10-month-old recording sessions. The average agreement between every two transcribers regarding the frequency of use of each potential VMS consonant (/p,b/, /t,d/, /k,g/, m, n, ŋ, l, s) was 69% (range 65–72%). Most disagreements had to do with the very infrequently used consonants, /l/ and /s/. The average agreement for all other consonants was 80% (range 76% to 89%). Given that the transcription was of prelinguistic babble, this degree of agreement is consistent with similar previous studies (e.g., Vihman et al., 1985; Davis and MacNeilage, 1995; McCune and Vihman, 2001).

#### Auditory Headturn Preference Paradigm

The stimuli were recorded using a female speaker with a Northern English accent, speaking in an infant-directed manner. All items were recorded in a sound-treated room (IAC Model 400) using a Sennheiser ME 66 microphone (with K6 power module) connected to a Tascam DA-P1 digital recorder sampling at 44.1 K Hz. The stimuli were transferred digitally onto a PC hard drive for eventual output. A multivariate ANOVA comparing amplitude, duration and mean F0 across the four word lists used in the familiar and rare conditions revealed no difference in any of these measures (*p*-values of 0.292 for amplitude, 0.512 for duration and 0.81 for mean F0).

The AHPP procedure used was similar to that described in Kemler Nelson et al. (1995). Seated on the caregiver’s lap in a quiet darkened sound-treated room, the infants faced the central panel of a three-sided test booth where a camera and red light were mounted. A blue light and speaker were mounted on each side panel. A PC and video monitor were located in the adjoining room where the experimenter controlled stimulus presentation and recorded infant LTs by pressing the left and right mouse buttons. The computer initiated and terminated trials in response to signals from the experimenter. In each trial, the infant’s gaze was centered by the blinking red light. The experimenter then initiated the computer trial by activating a blinking blue light to the left or right of the infant. When the infant was judged to orient to the blue light, a trial was presented from that speaker. If the infant looked away from the speaker for more than 2 s of accumulated time, the trial was terminated and another begun. Multi-talker babble created from the same speaker of the stimuli used in the experiment was delivered to the headphones worn by both experimenter and caregiver to mask the actual test stimuli. The caregiver also wore foam-insert hearing protection. All stimuli were presented at an average level of 65 dB (Tenma 72-6635 sound level meter).

Each experimental session consisted of an exposure and a test phase. In the exposure phase the infant was presented with two lists of each of the two test conditions, Familiar and Rare, counterbalanced for order such that half the infants heard Familiar first and half heard Rare. The exposure trial lists consisted of a randomized presentation of the 12 words. This condition was intended to expose the infant to the test procedures, since our previous experiments using the AHPP paradigm have indicated that the initial trials lead to overly long LTs that seem not to be indexed to the type of stimuli presented. In both exposure and test phases the word type was randomly assigned to either side.

The test phase of the experiment consisted of 12 trials, six each of the two test conditions. The words in the test trial lists were pseudo-randomized such that each pair of words appeared first in one trial. This ensured that each infant heard each of the 12 Familiar and 12 Rare words at least once, even if trials were terminated early. The order of presentation in the test phase was designed to ensure that the first four trials were counterbalanced across test conditions, such that they included two trials of each test condition, in varying orders, counterbalanced across infants. The counterbalancing at the beginning was designed to control for an anticipated decrease in LTs, independent of the stimuli, over the course of the test trials (see Vihman et al., 2004, for an analysis of LT by trial). The final eight trials were pseudo-randomized such that no more than two test trials of the same kind (Familiar or Rare) occurred in sequence. In both phases, the side of presentation was pseudo-randomized such that no more than two successive presentations from one side were allowed. The experiment lasted less than 10 min; the actual time was dependent upon the infant’s attention.

## Results

### AHPP Participants

Fifty-three of the infants tested on the AHPP completed the task. The results from six others were discarded due to suspected otitis media (*N* = 1), an eye condition which precluded judging direction of look (*N* = 1), experimenter error (*N* = 2) and excessive fussiness leading to early termination of the test (*N* = 2). The mean age at test was 309 days (*SD* = 4 days). Mean Age at 2 VMS for the 53 infants who completed the AHPP task was 313 days (*SD* = 41 days). The range was from 276 to 457 days (for infants who had not reached 2 VMS by the time of the AHPP, measuring continued after the AHPP until the 2-VMS point was reached.)

### Receptive Lexicon Size

The infants exhibited a steady growth in lexicon as measured by the CDI data (see **Figure 3**, below). As an important check on the AHPP experiments we correlated the mean number of words on the CDI that the infants were reported to know at 9 months (*M* = 12.3, *SD* = 11.9) with the mean number of stimulus words on the AHPP test that the infants were reported to know (*M* = 1.44, *SD* = 1.48). The two lexical measures were strongly correlated: *r* = 0.64 (*p* < 0.01), indicating that the words used in the AHPP provide a good sample of the first words comprehended.

### AHPP Looking Time Analysis

There was no difference between the Familiar (*M* = 5.71 s, *SD* = 2.05) and Rare LTs (*M* = 5.68 s, *SD* = 1.86); *t*(52) = 0.101, *p* = 0.92, *d* = 0.013 (correcting for the correlation, see Dunlap et al., 1996). To control for differences in individual infants’ attention span we base further analyses not on differences in mean LT but rather on the proportion of time an infant looked toward Familiar stimuli out of total LT to both Familiar and Rare:

Preference⁢ ratio =LT(Fam.)/[LT(Fam.)+LT(Rare)]

A value equal or close to 0.5 signifies no preference, values over 0.5 signify longer looking toward the Familiar stimuli (i.e., a familiarity preference) and values under 0.5 signify longer looking toward the Rare stimuli (a novelty preference).

**FIGURE 2:**
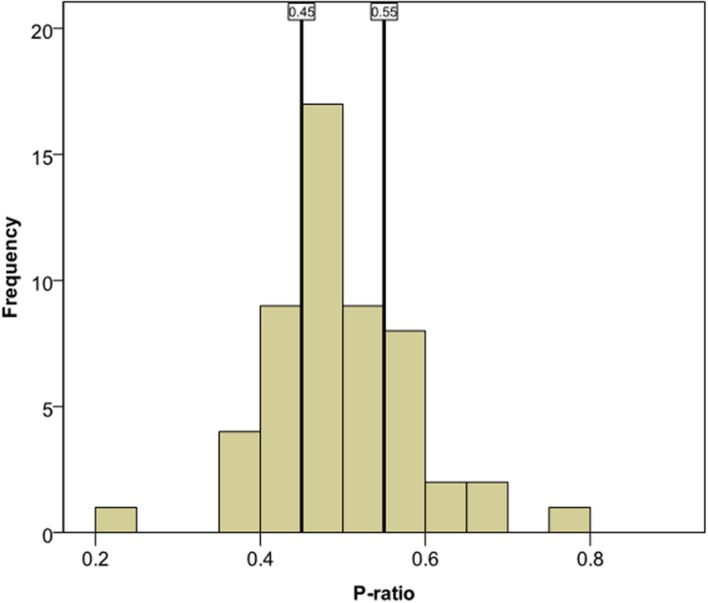
**Distribution of *p*-ratios for the AHPP.** Vertical lines show the cutoff points for the categorization of a score as extreme or moderate. *P*-ratio scores to the left of the 0.45 line or to the right of the 0.55 line are ‘extreme’ and are classified as novelty and familiarity preferences respectively.

As can be seen in **Figure 2**, the preference ratio (*p*-ratio) distribution is normal [Kolmogorov–Smirnov (53) = 0.107, *p* = 0.185], as expected: see discussion above. The distribution is centered on the no-preference value of 0.5 and displays both extreme familiarity and extreme novelty responses. We divided the *p*-ratios such that both ends of the scale, novelty and familiarity preference, would be taken to signify ‘success’ in the task while the middle portion, no preference, would signify ‘failure.’ The criterial point for distinguishing between ‘Pass’ and ‘Fail’ was chosen so as to create two similar-sized groups, with sample sizes of 26 and 27, respectively. The half of the *p*-ratio scores which were furthest away from 0.5 (in either direction) were considered a ‘Pass,’ and the half which were closest to 0.5 (in either direction) were considered a ‘Fail.’ The cutoff point for this binary scale turned out to be a distance of 0.0501 from the 0.5 point on the *p*-ratio scale: *P*-ratio values above 0.5501 or under 0.4499 were classified as extreme (‘Pass’) and those between those two values were classified as moderate (‘Fail’)^2^ (see **Figure 2**).

In order to test whether this criterion for dividing the group into two is simply a proxy for age, such that the extreme group is older than the moderate group, we compared the two groups on average age. Recall that our hypothesis is that the infants showing either a novelty or a familiarity preference are more linguistically advanced, not simply older. An independent *t*-test showed that the mean ages for the two groups do not differ [M (Moderate) = 308.9 days, *SD* = 4.0, M (Extreme) = 309.9 days, *SD* = 4.1, *t*(df = 51) = -0.928, *p* = 0.36] and their ranges and standard deviations are very similar.

### Testing the Relationship between the AHPP and Language Advance

#### Performance on the AHPP and Infants’ Receptive Lexicon Size

First we tested the relationship between the AHPP and lexicon (as measured on the CDI at 9 months). Because the distribution of lexicon size by preference type is skewed, we assessed its goodness of fit to both a Poisson and Negative binomial distribution. Only the Negative Binomial turned out to be a good fit (Goodness of fit ratio (value/df) of 1.239, *p* > 0.1). Thus we ran a Generalized Linear Model (GLM) using the Negative Binomial distribution with a log link, with the binary AHPP score as a predictor and number of words on the CDI as a predicted variable. The mean number of words known by the infants was *M* = 15.7 for the Extreme group (novelty and familiarity grouped together) and *M* = 8.9 for the No-Preference group. The GLM was very close to being significant (Wald χ^2^ = 2.690, df = 1, *p* = 0.051, one-tailed, η^2^ = 0.79, where η^2^ is calculated assuming normality). Thus, the infants who exhibited either a familiarity or a novelty preference tended to know more words than those who showed no preference. We chose a one-tailed test since our original hypothesis was that the infants with familiarity and novelty responses would have a larger lexicon. The alternative possibility, that infants with no preference on the AHPP would have higher scores on the CDI, is not consistent with the Hunter and Ames (1988) model or in fact with any model of infant response to novel or familiar speech stimuli.

One possible reason for the failure of the GLM to achieve statistical significance is the fact that only 36 parents filled out the CDI at 9 months of age, which led to a reduction in the power of the test. The large effect size (η^2^ = 0.79) supports this and suggests that the difference might be significant with a larger sample. This limitation in sample size was also the reason that we ran the test as two groups (Novelty/Familiarity versus No preference) instead of three (Novelty versus Familiarity versus No preference), as our original hypothesis would suggest.

**FIGURE 3:**
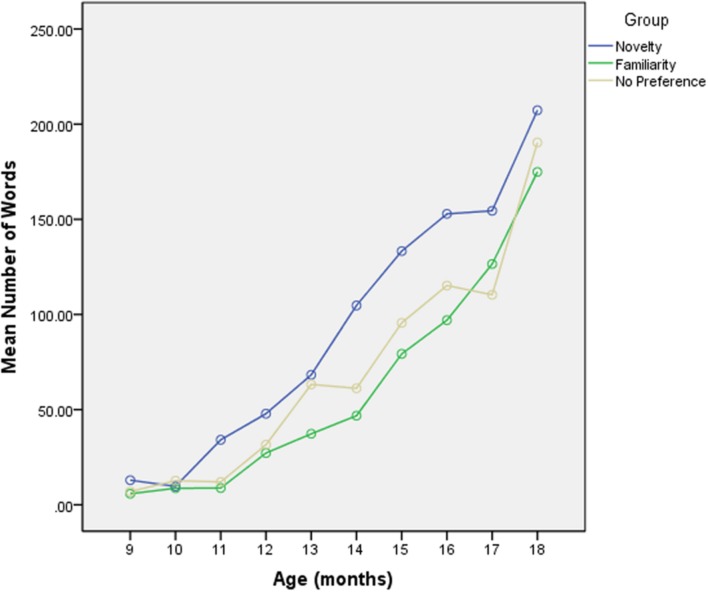
**The average growth on the CDI by response on the AHPP (low, mid, and high preference ratio infants)**.

To begin to test our hypothesis directly (and in effect to test the Hunter and Ames model) we first plotted lexical growth for each group (see **Figure 3**). Here we can see that the Novelty group separates out from the Familiarity and No-preference groups, the latter two being indistinguishable.

To test our hypothesis that the preference exhibited in the AHPP is indicative of lexical advance as measured by the CDI we used a fixed effects model with a first order autoregressive covariance structure that assumed the repeated measures (CDI across 10 months) were correlated within each infant but independent across infants. The results show both group [*F*(2,107.8) = 5.927, *p* = 0.004] and age [*F*(1,195.8) = 265.894, *p* < 0.001] to be significant, with no difference between the Familiarity and No-preference groups and a significant difference between the Novelty and the No Preference group (see **Figure 3**; **Table 2**).

**Table 2:** Fixed effects model results.

Parameter	Estimate	Standard error	df	*t*	Significance
Intercept	-182.56	16.00	183.44	-11.41	0.000
Novelty	27.40	8.75	107.27	3.13	0.002
Familiarity	-2.06	9.14	108.52	-0.23	0.822
No preference^∗^	0	0			
Age	18.51	1.14	195.84	16.31	0.000


*^∗^This parameter is set to zero since it is redundant.*

#### Performance on the AHPP and Infants’ Phonetic Advance (Age at Two VMS)

**FIGURE 4:**
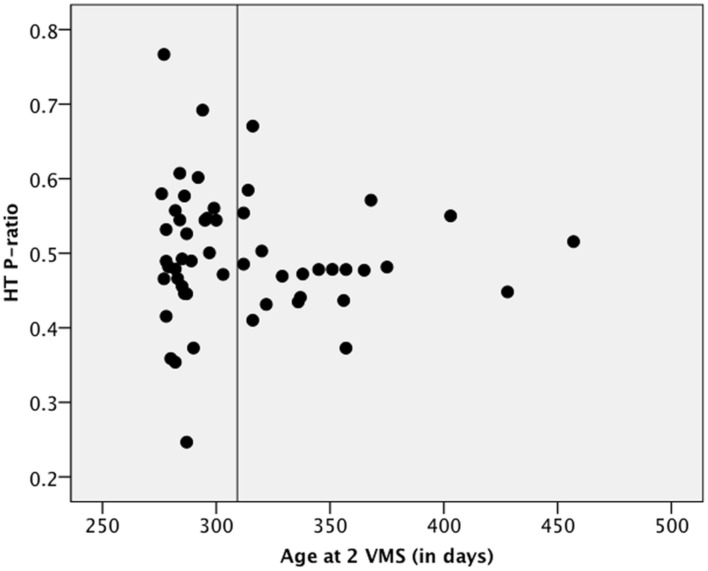
**Variability in *p*-ratios on the AHPP and VMS.** This variability reflects differences between infants who had versus had not attained two VMS by the test date. The vertical line represents the AHPP test date of 10 months. Infants to the left of the line had acquired two VMS by the AHPP test, while those to the right had not.

**Figure 4** plots the age at two VMS (in days) against *p*-ratios on the AHPP. The vertical line shows the average age, around 10 months, at which the infants were tested on the AHPP. The points to the left of the vertical line are the *p*-ratios of the infants who had attained two VMS by the day of their AHPP and those to the right of the line are those of the infants who had not yet attained two VMS by the test date. As can be seen, the *p*-ratios of the infants whose production is more advanced at the time of the test are much more widely dispersed than are those of their less advanced counterparts. The difference in variance between the group of infants who had not attained two VMS versus that of those who had is significant (Levene’s Test for Equality of Variances: *F* = 5.059, *p* = 0.029, df = 51). The greater dispersion in the group with the more advanced production stems from their having more extreme *p*-ratios than the infants who have not yet attained two VMS. Thus, the infants who are more advanced phonetically are also more likely to show either a strong novelty or a strong familiarity effect.

## Discussion

The findings of this study indicate that the infants who show either a strong novelty or a strong familiarity response indeed make up the more linguistically advanced group: strong preference for either type of stimulus was seen in the infants who attained two VMS earlier but not in those who reach that level of phonetic mastery only later (**Figure 4**). This figure is interesting from at least two perspectives. First, it implies that phonetic advance in production is connected to success on the AHPP in recognizing words. This is supported by three recent studies that have found a correlation between vocal production and speech perception in prelinguistic infants (DePaolis et al., 2011, 2013; Majorano et al., 2014) and another study that found that obstructing the tongue of prelinguistic infants impaired their ability to discriminate phonemes whose production involves movement of the obstructed tongue (Bruderer et al., 2015).

Second, it suggests that variability in this word recognition task changes developmentally, with stability in responses decreasing as infants became more advanced phonetically and lexically. Importantly, this advance is not necessarily tied to age but instead to each infant’s individual developmental path. The variability in **Figure 4** is reminiscent of Thelen and Smith’s (1994) claim that transitions from one stable state (in this example, an inability to recognize word forms) to another (the ability to recognize often-heard word forms) is characterized by instability. Thus, **Figure 4** indirectly supports a dynamic systems approach to early lexical development (see Vihman et al., 2016, where that approach is related to early phonological development).

Finally, analysis of the CDI at 10 months of age (see Performance on the AHPP and Infants’ Receptive Lexicon Size), the age of the infants when tested on the AHPP experiment, lent partial support to a Hunter-and-Ames-type model as the AHPP results tend to differentiate the infants by lexical advance. This finding is presented with caution since a one-tailed test just missed reaching significance *and* we could test only the no-preference group versus the combined familiarity and novelty groups. We now take up AHPP and lexical advance in more detail, and in the process, test the Hunter and Ames model more directly.

### AHPP Looking Time Analysis: The Distribution

As we expected, the distribution of preferences in the AHPP was normal around a *p*-ratio of 0.5, reflecting equal interest in both the familiar and unfamiliar words. Interestingly, if we had not collected independent indices of phonetic and lexical advance we might have reported this as a null finding and concluded that 10-month-old infants show no sign of recognizing word forms. This in fact is what Vihman et al. (2007) concluded when they used this paradigm with 10-month-olds.

There is growing evidence, however, that studies that are indexed to chronological age are at risk of null findings when the data are actually masking developmental change. For example, recent studies examining electrophysiological responses to VOT distinctions in English infants found a similar null effect that turned out to be a mixture of different levels of advance, similar to what we found in this study. Rivera-Gaxiola et al. (2005a,b) examined the responses of 7- and 11-month old infants to both native and non-native language differences in VOT. They found that while 7-month-old infants’ event related potentials (ERP) were discriminatory between native and non-native VOT contrasts, there was no significant difference in the ERP patterns to the non-native contrasts at 11 months of age. Upon closer examination of the results, however, two groups of infants emerged: One group of 11-month old infants displayed larger amplitude in the N250–550 response to *both* native and non-native contrasts. The other group displayed a differential response, a larger amplitude N250–550, for the native contrasts only, along with a larger P150–250 response to the non-native contrast. The authors interpreted this latter response as being more mature, possibly suggesting that the infants experienced the non-native contrast as irrelevant, which allowed them to disregard it. That this latter group was indeed more linguistically advanced is also supported by the fact that its members had larger receptive vocabularies from 18 to 30 months of age.

Another ERP study by Kooijman et al. (2013) found that 7-month-olds exhibited an overall positive right frontal and negative left posterior response to familiar words when they were embedded in sentences. In this case the authors also found two subgroups of infants: The majority showed a positive familiarity response on right frontal electrodes; a smaller number exhibited a negative left frontal and posterior response at 7 months, similar to that demonstrated by 10-month olds in a previous study (Kooijman et al., 2005). This minority group also had significantly higher scores on word comprehension and on sentence and word production at age three.

Thus, similar to the research by Rivera-Gaxiola et al. (2005a,b) and the current study, Kooijman et al. (2005, 2013) found that two patterns of responses could be identified and explained once the results were referenced to other measures of linguistic advance. In all of these studies, without the additional measures of linguistic advance, the group results would have masked important differences, highlighting that experiments conducted at a set chronological age are likely to include subgroups of infants that cover a range of different developmental stages. In both electrophysiological studies data clearly separate the infants into groups that differ in measures of language advance when examined years later. Similarly, the infants who show a familiarity or novelty response in the current experiment proved to be more advanced when we examine the AHPP results closely in relation to the lexical growth data from the CDI. We now consider the clearest evidence for this.

### Performance on the AHPP and Infants’ Receptive Lexicon Size

The growth in receptive lexicon began earlier for the Novelty group (see **Figure 3**) but there was no difference between the Familiarity and No-preference groups. Hunter and Ames’ (1988) model predicts a difference in LTs between the Familiarity and the No-preference groups but the actual difference between the two groups does not seem to translate here to lexical growth. So why is the CDI growth rate of the No-preference and Familiarity infants the same? It may be that the infants who are not showing a preference are on the cusp of this advance. The group effect of word form recognition is robust at 11 months of age (Hallé and de Boysson-Bardies, 1994, 1996; Vihman et al., 2004; Swingley, 2005; Vihman et al., 2007), only a month after the age at which the infants were tested in this study. So, while the Novelty group may be truly advanced, the No-preference and familiarity groups could be developmentally much more similar. In addition, if there truly is a developmental shift from familiarity to novelty, it is possible that some of the infants, if tested while in this transition period, will therefore exhibit no preference (but see the discussion in the introduction, above).

We suggest that another reason to suspect that a novelty response indicates a more advanced level of language processing is the robustness of the representation of the word that is required for an infant to exhibit such a response. A novelty or familiarity response indicates that the infant has maintained a memory of the stimulus that has lasted from the time when the infant was last presented with it until it is presented in the experiment (see the discussion in Civian et al., 2005, regarding novelty responses to visual stimuli elicited from 16-week-old infants). In the current study, the stimuli are words commonly heard in everyday situations, before the infant is brought to the lab. For a novelty response, memory for the words must be robust enough to render these familiar words too well established to warrant attention. Just the opposite effect underlies a familiarity response; representations for these words are just beginning to form in memory and are thus of interest in themselves (cf. Kidd et al., 2012, who showed that infants’ attention to visual stimuli is increased when the stimuli are neither too complex nor too simple). Thus, while the familiarity responses indicate the beginning of word-form recognition, a novelty response suggests that the infants have stable memory representations of words. It is possible that those infants are also beginning to associate meaning with these words.

### Summary

This paper presents a novel approach to the analysis and interpretation of group results on the AHPP. Its implications are particularly pertinent for cases in which the responses to an AHPP are distributed symmetrically around the no-preference value, resulting in a lack of preference for either type of stimulus at the group level. Our findings show that such results may still be informative at the subgroup level. Such a distribution may be indicative of a mixed group, containing both advanced infants, who have successfully distinguished between the two types of stimuli, and less advanced infants, who have not. Crucially, in order to interpret such results, additional measures of advance in a related cognitive domain must be independently obtained for the same infants. It should then be possible to determine whether the lack of a group effect is due to individual infants not distinguishing between the stimuli or to the ability of different infants to make such a distinction, as manifested in different preferences. With regards to our initial question as to the suitability of the Hunter and Ames Model for word learning, we have found evidence that, as regards word-form recognition, no-preference, familiarity and novelty responses do seem to reflect different stages of advance in the independent domains of vocal production and lexical comprehension. The familiarity subgroup patterns with the more advanced novelty subgroup in vocal production but with the less advanced moderate group in lexical advance. Our data therefore provide only partial support for a Hunter and Ames model of word form recognition. Future studies, if they can achieve greater precision in measuring infants’ lexical knowledge, may be able to show clearer separation between the groups exhibiting these three types of responses.

## Author Contributions

RD, MV, and TK-P all wrote the grant that funded this project. In addition, they all helped to interpret the findings. RD wrote the paper while TK-P was instrumental in managing the researchers who collected the data (although all authors were involved).

## Conflict of Interest Statement

The authors declare that the research was conducted in the absence of any commercial or financial relationships that could be construed as a potential conflict of interest.

## Footnotes

1 This idea was proposed 16 years ago at the 12th Biennial International Conference on Infant Studies (Aslin, 2000), and was recently advanced in a symposium at the 17th Biennial International Conference on Infant Studies (Dawson and Kidd, 2010).

2 The distribution can also be divided differently, with p-ratios in both the lowest and the highest quartiles being treated as success and those in the two middle quartiles as failure. The two methods of categorization result in nearly identical groups, with only three infants (out of a total of 53) being placed in different categories. It is worth noting that there are a number of ways to sort the infants into three groups. For example, from a statistical standpoint it could be argued that it would be best to create equal group sizes to increase the numbers in the novelty and familiarity groups, and thus potentially increase the power of the test (as proposed by one reviewer). We did not use this option since there is no reason to assume that a sample of 59 infants would include equal numbers of infants exhibiting novelty and familiarity preferences. We chose instead to minimize the chances that infants who have no preference for either word type would end up classified in the novelty or familiarity group. This was accomplished by classifying only the most extreme 50% of the infants as exhibiting a preference.
